# Mutational analysis of immunodominant epitopes of caprine β-casein recognized by IgE antibodies from patients allergic to goat’s milk and tolerant to cow’s milk

**DOI:** 10.1186/2045-7022-3-S3-O11

**Published:** 2013-07-25

**Authors:** S Hazebrouck, S Ah-Leung, E Bidat, E Paty, M-F Drumare, S Tilleul, K Adel-Patient, J-M Wal, H Bernard

**Affiliations:** 1 Laboratoire d'Immuno-Allergie Alimentaire, INRA, Gif-sur-Yvette, Paris, France; 2 Service de Pédiatrie, AP-HP, Hôpital Ambroise Paré, Boulogne-Billancourt, France; 3 Université Paris Descartes, AP-HP, Hôpital Necker Enfants Malades, Paris, France

## Background

Several cases of allergy to goat's milk (GM) without allergy to cow's milk (CM) have been reported. GM-allergy has also been reported in CM-allergic children successfully treated with oral immunotherapy. We previously demonstrated that IgE antibodies from GM-allergic/CM-tolerant patients recognize the caprine β-casein (βcap) without cross-reacting with the bovine β-casein (βbov) despite a high sequence identity (91%). We aimed in the present work to identify the critical amino acids in the non-cross-reactive IgE-binding epitopes of βcap.

## Methods

Using site-directed mutagenesis, recombinant βcap was modified by performing residue substitutions with the corresponding amino acids found in βbov. The IgE-binding capacity of the different modified βcap was then evaluated with sera from 9 GM-allergic/CM-tolerant patients and 9 CM-allergic patients. The specificity of murine monoclonal antibodies (mAb) raised against caprine caseins was also analyzed in order to further characterize non-cross-reactive epitopes. The allergenic activity of recombinant βcap was finally assessed by degranulation tests of RBL cells passively sensitized with human IgE antibodies.

## Results

The substitutions A55T/T63P/L75P in the N-terminal part and P148H/S152P in the C-terminal part of βcap induced the greatest decrease of IgE-reactivity of GM-allergic/CM-tolerant patients toward the caprine allergen. The threonine 63 was found to be particularly critical, as confirmed by the specificity of mAb SCB1D, whose ability to bind βcap was abolished by the substitution T63P. The recombinant βcap containing the five substitutions was unable to induce the degranulation of RBL cells passively sensitized with IgE from GM-allergic/CM-tolerant patients but was still fully allergenic when testing sera from CM-allergic patients.

## Conclusion

Most of the critical substitutions supporting the restricted IgE specificity of GM-allergic/CM-tolerant patients toward βcap involved proline residues. This probably affects both the primary and secondary structures of non-cross-reactive epitopes since proline are frequently found in turns in protein structures. The drastic influence of substitution T63P on the binding of mAB SCB1D to βcap confirmed the immunodominant role of the epitope encompassing threonine 63, as initially observed with GM-allergic/CM-tolerant patients.

## Disclosure of interest

None declared.

